# A novel and improved selective media for the isolation and enumeration of *Klebsiella* species

**DOI:** 10.1007/s00253-022-12270-w

**Published:** 2022-11-16

**Authors:** Megha Prasad, Sindhu K. Shetty, Bipin G. Nair, Sanjay Pal, Ajith Madhavan

**Affiliations:** School of Biotechnology, Amrita Vishwa Vidyapeetham, Kerala, 690525 India

**Keywords:** Wastewater surveillance, Selective medium, *Klebsiella* spp., Differential medium

## Abstract

**Abstract:**

Bacterial pathogens are fostered in and transmitted through wastewater. Hence, monitoring their impact on sanitation and hygiene is imperative. As part of the monitoring process, culture-based methodologies are primarily used, which centre on the use of selective and differential media. Media available today are, at best, difficult to formulate and, at worst, prohibitively expensive. To address this lacuna, the study proposes a selective and differential medium for *Klebsiella* spp. *Klebsiella* blue agar (KBA) is completely selective against selected gram-positive bacteria (*Bacillus* spp., *Staphylococcus aureus*) and a few gram-negative bacteria (*Acinetobacter baumanii*, *Serratia marcescens*)*.* On the other hand, it supports the growth of the chosen members of the *Klebsiella pneumoniae* species-complex with a characteristic green colouration. Methylene blue, tryptophan, and bile salt make up the selective components of KBA. Moreover, methylene blue, 0.6% NaCl, and glycerol render it differential. KBA was more selective than HiCrome™ Klebsiella Selective Agar Base (KSA) in replica plating experiments. KBA promoted only 157 CFUs against 209 CFUs in KSA when stamped with 253 CFUs grown on LB. The colonies so isolated were predominantly *Klebsiella* spp., on identification through colony polymerase chain reaction. Moreover, the differential nature of KBA distinguished *Klebsiella aerogenes* from other species. On the contrary, KSA lodged colonies indistinguishable from each other and *Klebsiella* spp. Due to its ease of formulation, high selectivity, differential nature, and cost-effective composition, KBA is a viable option for the routine culture of *Klebsiella* spp. in environmental and clinical settings.

**Key points:**

• *Formulated a novel selective and differential media for Klebsiella spp., named Klebsiella Blue agar*

• *Facile formulation methodology*

• *Can be employed to isolate Klebsiella spp. from complex sources such as wastewater*

**Supplementary information:**

The online version contains supplementary material available at 10.1007/s00253-022-12270-w.

## Introduction

Pathogens of public health importance transmitted through direct or indirect contact between humans, animals, and the environment are the leading cause of emerging and re-emerging infectious diseases all over the globe (Galarde-López et al. 2022). These infectious diseases have an adverse impact on global economies and public health (Jones et al. 2008). Amongst these infectious agents, the *Enterobacteriaceae* family has been fast gaining attention as this group has been linked to a high percentage of hospital-acquired infections, and most antibiotics are often ineffective against them (Babu et al. 2016; Sakkas et al. 2019; Rolbiecki et al. 2021). *Klebsiella* genus, a class of gram-negative, encapsulated, non-motile bacteria belonging to the *Enterobacteriaceae* family (Dworkin et al. 2006; Grimont and Grimont 2015; Wyres et al. 2020) is one of the leading causes of nosocomial and community-acquired infections. *Klebsiella* spp. is grouped into cohorts, namely *Klebsiella pneumoniae* species complex (KpSC), which includes *Klebsiella pneumoniae*, *Klebsiella quasipneumoniae*, and *Klebsiella variicola*, while *Klebsiella oxytoca*, *Klebsiella indica*, and *Klebsiella terrigena* (Dong et al. 2022) into another genetically distinct group. The KpSC group of bacteria is responsible for most nosocomial and community-acquired pneumonia, urinary tract, and bloodstream infection associated with *Klebsiella* spp. in healthcare-associated settings (Prado et al. 2008; Stojowska-Swędrzyńska and Krawczyk 2016; Martin and Bachman 2018; Dong et al. 2022). These bacteria can thrive in various niches, including plants, animals, and waterbodies (Holt et al. 2015). They have an uncanny ability to exchange their plasmid with other species. This property and high genomic plasticity make these species a reservoir of virulence and antimicrobial resistance genes (Ramirez et al. 2014). The World Health Organisation in 2017 declared the extended-spectrum β-lactam (ESBL)-producing and carbapenemase-producing *Klebsiella* spp. a latent threat to public health due to its ability to accumulate multidrug resistance (MDR) and hypervirulence (Zhou et al. 2016), especially in wastewater which is a hotbed for acquiring and disseminating MDR genes (Moges et al. 2014; Gomi et al. 2018; Bonardi and Pitino 2019; Perez-Palacios et al. 2021a).

Nutrient-rich wastewater and waterbodies where the bacterial cell density is exceptionally high, the factors influencing the increase in antibiotic resistance in bacteria are enhanced; for example, hospital effluents are an ideal pool for exchanging resistance genes between clinical and environmental bacteria (Sakkas et al. 2019). Outside of the clinical settings, little is known about the ecology and transmission of *Klebsiella* spp.; hence, the detection, identification, and monitoring of *Klebsiella* spp. and their different clonal groups in the environment and effect on humans remain undefined (Mathers et al. 2015; Holt et al. 2015). Understanding the emergence and spread of these antibiotic-resistant bacterial strains in the environment requires wastewater-based epidemiological monitoring and surveillance system (Hornsey et al. 2013; Daughton, 2020; Galarde-López et al. 2022). Molecular DNA-based techniques like pulsed-field gel electrophoresis, multilocus sequence typing, repetitive element sequence-based PCR, and whole genome sequencing (Dinkelacker et al. 2018) are currently employed for this purpose. However, being laborious and cost-limiting, these high-end techniques are restricted to research rather than routine real-time surveillance (Rossen et al. 2018).

Developing a cost-effective, easy-to-formulate, selective, and differential bacterial culture media is imperative to make wastewater monitoring and surveillance more rigorous and hassle-free. A primary medium is rendered selective and differential by adding components such as dyes, chemicals, and antibiotics. However, rising antibiotic resistance and lack of exploration of new chemical additives as selective agents have curtailed the development of a new and improved selective medium. Over the years, many different selective culture methods have been proposed for active surveillance of *K. pneumoniae* and its associated clonal groups in different settings. These include but are not restricted to — MacConkey agar supplemented with ceftazidime, *Klebsiella ChromoSelect* Selective Agar Base, Simmons citrate agar (SCA) with 1% inositol, and HiCrome™ *Klebsiella* Selective Agar Base (van Kregten et al. 1984; Glupczynski et al. 2007; Charles et al. 2022). However, these media owing to their prohibitive cost, formulation complexity, and indistinguishable nature towards KpSC and other *Klebsiella* strains are not extensively used for routine surveillance of wastewater. Thus, the need of the hour is to develop a selective and differential medium that selectively grows and differentiates between the species belonging to the KpSC group and other genetically distant species of *Klebsiella*.

In this study, we sought to evaluate a novel medium termed *Klebsiella* blue agar in selectively promoting the growth of *Klebsiella* spp. and differentiating the members of the two cohorts. The components of the proposed medium are readily available and rationally put together to render it selective and differential. KBA was compared with KSA’s ability to promote and differentiate the species. Also, compared to the KSA, the discriminatory power of the KBA medium in differentiating the *Klebsiella* sp. belonging to the group KpSC from other enteric bacteria for potential integration into routine surveillance workflow was studied using simulated sewage as the source of the environmental sample.

## Materials and methods

### Media

The composition of *Klebsiella* blue agar media per litre of distilled water is as follows: solution A — 3 g potassium dihydrogen phosphate (Sisco Research Laboratories Pvt. Ltd.), 6 g dipotassium phosphate (EMPLURA, Merck Life Science Pvt. Ltd.), 6 g sodium chloride (Sigma Aldrich), 64 mg methylene blue (Spectro Chem Pvt. Ltd.); solution B — 100 mg magnesium sulphate dihydrate (MERCK Specialties Pvt. Ltd.) and 17 g agar (Hi Media Laboratories Pvt. Ltd.). Solutions A and B were separately autoclaved at 121 °C for 20 min. On cooling, filter sterilised bile salt, tryptophan, and glycerol 1.5 g/L, 2 g/L, and 0.2%, respectively, were added to solution A. KBA was constituted by mixing solutions A and B and poured onto sterile Petri dishes. The Luria Bertani broth (LB) (Hi Media Laboratories Pvt. Ltd.) and HiCrome™ *Klebsiella* Selective Agar Base (HiMedia Laboratories Pvt. Ltd.) were used for comparison.

### Bacterial cultures

Multidrug-resistant clinical strains of *Klebsiella pneumoniae* K2, *Klebsiella pneumoniae* K3, *Klebsiella pneumoniae* K4, *Klebsiella pneumoniae* K5, *Klebsiella pneumoniae* U4677, *Klebsiella pneumoniae* U4698, *Klebsiella pneumoniae* U4865, and *Klebsiella pneumoniae* OF9168 were gifted by Dr Anil Kumar, Head of the department, Department of Microbiology, School of Medicine, Amrita Vishwa Vidyapeetham, Kerala, India. The clinical strains of *Shigella dysenteriae*, *Salmonella enterica*, *Klebsiella quasipneumoniae*, and *Vibrio cholerae* were gifted by Dr Bhabatosh Das, Associate Professor, Translational Health Science and Technology Institute, Delhi, India. *Acinetobacter baumannii* (MTCC 1425), *Klebsiella pneumoniae* (MTCC 3384), *Pseudomonas fluorescens* (MTCC 1749), and *Serratia marcescens* (MTCC 97) were procured from the Microbial Type Culture Collection and Gene Bank, Chandigarh, India. The laboratory strains of *Proteus vulgaris*, *Staphylococcus aureus*, and *Klebsiella aerogenes* were obtained from the Academic Laboratory of the School of Biotechnology, Amrita Vishwa Vidyapeetham, Kerala, India. The isolates of *Bacillus* spp. and *Pseudomonas putida* were isolated from soil, while *Escherichia coli* sequence type 155 (Salim et al. 2019) and *Klebsiella variicola* (Subhash et al. 2022) were isolated from sewage at the Sanitation Biotechnology Lab, School of Biotechnology, Amrita Vishwa Vidyapeetham, Kerala, India. All the bacterial strains were maintained in Luria–Bertani (LB) at 37 °C. In broth cultures, bacterial strains were grown in LB broth at 37 °C with 200 rpm.

### Growth characteristics on *Klebsiella *blue agar

To assess the growth, respective cultures of *Klebsiella* spp. were streaked on the KBA media. The KBA plates were incubated at 37 °C for 24 h. The growth and morphology of the bacterial cultures in KBA after 48 h were also studied. The tests were performed independently and in duplicate.

### Selective and differential nature of KBA

All the available bacterial strains were grown overnight at 37 °C in LB broth and were used to check the ability of KBA to differentiate *Klebsiella* spp. from other enteric/non-enteric bacteria. The KBA media were compared to the commercial media — KSA, which is selective for *Klebsiella* species. Since all the selected bacterial cultures grow efficiently in LB, it was used as general media control. The cultures were streaked onto KBA, KSA, and LB agar plates and incubated for 24 h at 37 °C. Extended incubation of the plates was done for 48 h at 37 °C. The tests were performed independently and in duplicate.

### Efficiency evaluation of KBA in surveillance of *Klebsiella *spp. in synthetic sewage

The efficiency of the KBA media in surveillance of sewage for the presence of *Klebsiella* spp. was checked in synthetic sewage augmented with bacteria as described in the literature (Salim et al. 2022) with modifications. Peptone, 160 mg; meat extract, 110 mg; anhydrous dipotassium hydrogen phosphate (K_2_HPO_4_), 28 mg; sodium chloride (NaCl), 7 mg; calcium chloride dihydrate (CaCl_2_.2H_2_O), 4 mg; urea 30 mg; and magnesium sulphate heptahydrate (MgSO_4_.7H_2_0), 2 mg were added to 1 L water and autoclaved. Chemical characteristics of the synthetic sewage were estimated as per the Indian Standard 3025 (IS 3025) (IS 3025 of Indian Standards Part 11, IS 3025 Part 44, IS 3025 Part 38, IS 3025 Part 16), as reported in the literature from the laboratory of the authors (Salim et al. 2022). Synthetic sewage was augmented with the following cultures at an OD of 0.1 each, *K. pneumoniae*, *K. quasipneumoniae*, *K. aerogenes*, *E. coli* ST155, *B. clausii*, *A. baumanii*, *S. enterica*, *P. vulgaris*, *V. cholerae*, *Shigella* spp., MDR strains *K. pneumoniae* OF9168, *K. pneumoniae* K2, *K. pneumoniae* K3, *K. pneumoniae* K4, *K. pneumoniae* K5, and *K. pneumoniae* U498. The augmented synthetic sewage was incubated for 1 h in a shaking incubator at 37 °C, following which the sample was plated at different dilutions onto the KBA, KSA, and LB media plates in triplicates and incubated at 37 °C for 24 h. Differences in the bacterial growth on all three media were analysed along with their respective CFU/mL. One-step multiplex colony PCR of the isolated colonies using the primers specific for *K. pneumoniae* and *K. quasipneumoniae* (Table 1) was used to evaluate the differential and selective nature of all three media. Bacterial colonies with different morphologies were selected for the molecular analysis. Colonies were resuspended in 100 µL of sterile water and pre-treated at 90 °C for 30 min before adding primers and reagents. PCR amplification conditions were optimised and performed as described by researchers (Fonseca et al. 2017). Briefly, thermocycling conditions were as follows: 95 °C for 5 min, followed by 40 cycles at 94 °C for 30 s, 62.5 °C for 30 s, 72 °C for 1 min, and a final extension step at 72 °C for 10 min. The amplified PCR products were analysed by electrophoresis on 2.5% agarose gel at 50 V for 2 ½ h and visualised using a gel doc system (Bio-Rad, USA). The amplicon size was determined using a 100 bp molecular weight marker (Origin Diagnostics and Research).

**Table 1:** Primers for identification of different *Klebsiella* species

Primer name	Primer sequence (5′-3′)	Size (bp)	Bacterial culture
SHV-F	GCTGGCGGTACACGCCAGCCCG	995	*Klebsiella pneumoniae*
OKP-F	GGCCGGYGAGCGGGGCTCA	348	*Klebsiella quasipneumoniae*
DeoR-R*	AGAAGCATCCTGCTGTGCG		

*The forward primers SHV-F and OKP-F were combined with the DeoR-R reverse primer in the multiplex PCR, producing the amplicons size as indicated in the column (Fonseca et al. 2017)

### Comparative study for determining the selectivity of KBA over KSA

Synthetic sewage augmented with the bacterial cultures was incubated for 1 h, as described in the previous section. The sample was drawn from the experimental set-up, serially diluted from 10^−1^ to 10^−8^, and plated onto LB agar plates. The plates were incubated overnight at 37 °C and replica plated onto KSA and KBA agar plates. A Sterile Whatman filter paper no. 1 of diameter 8.2 cm was used to replica transfer the colonies in the LB to the KBA and KSA agar plates. The plates were incubated overnight at 37 °C. The differences in CFU in different media were recorded.

### Statistical analysis

Statistical analysis was performed using the platform Graph Pad Prism 9.0. The data obtained were analysed using the one-way ANOVA test with Dunnett’s multiple comparison tests to indicate any statistical significance between LB, KBA, and KSA. The significance level for the data sets was defined at *p* ≤ 0.05. All the data sets in the graphs are presented as mean values with respective standard deviations.

## Results

### Media formulation

The components in KBA were judiciously included to render it selective and differential. Methylene blue, an inhibitor of the gram-positive organisms, was used to dissuade their growth in the medium. Bile salt and tryptophan were included as they are conducive to the growth of *Klebsiella* spp., leveraging on their ability to tolerate bile salt and metabolise tryptophan. Glycerol provided in the media acted as a sole carbon source suitable for *Klebsiella* spp. A total of 0.3% of NaCl rendered the media differential by imparting a dark green colouration to the *Klebsiella* spp. colonies (Table 2).

**Table 2:** Comparison of *Klebsiella* blue agar with the HiCrome™ *Klebsiella* Selective Agar Base (HiMedia Laboratories Pvt. Ltd.; https://himedialabs.com/TD/M1573.pdf)

Components	*Klebsiella* blue agar	HiCrome™ *Klebsiella* Selective Agar Base
Carbon source	Glycerol	Yeast extract
Nitrogen source	Tryptophan	Peptone special
Buffering agent	Potassium dihydrogen phosphate; dipotassium phosphate	-
Osmolarity	Sodium chloride	Sodium chloride
Micronutrient	Magnesium sulphate dihydrate	-
Selective component	Methylene blue; bile salt; tryptophan	*Klebsiella* Selective Supplement (FD225)—carbenicillin (25 mg); chromogenic mixture; bile salt mixture; sodium lauryl sulphate
Gelling agents	Agar	Agar

### *Colony morphology of Klebsiella spp. on the Klebsiella blue agar*

**Fig. 1:**
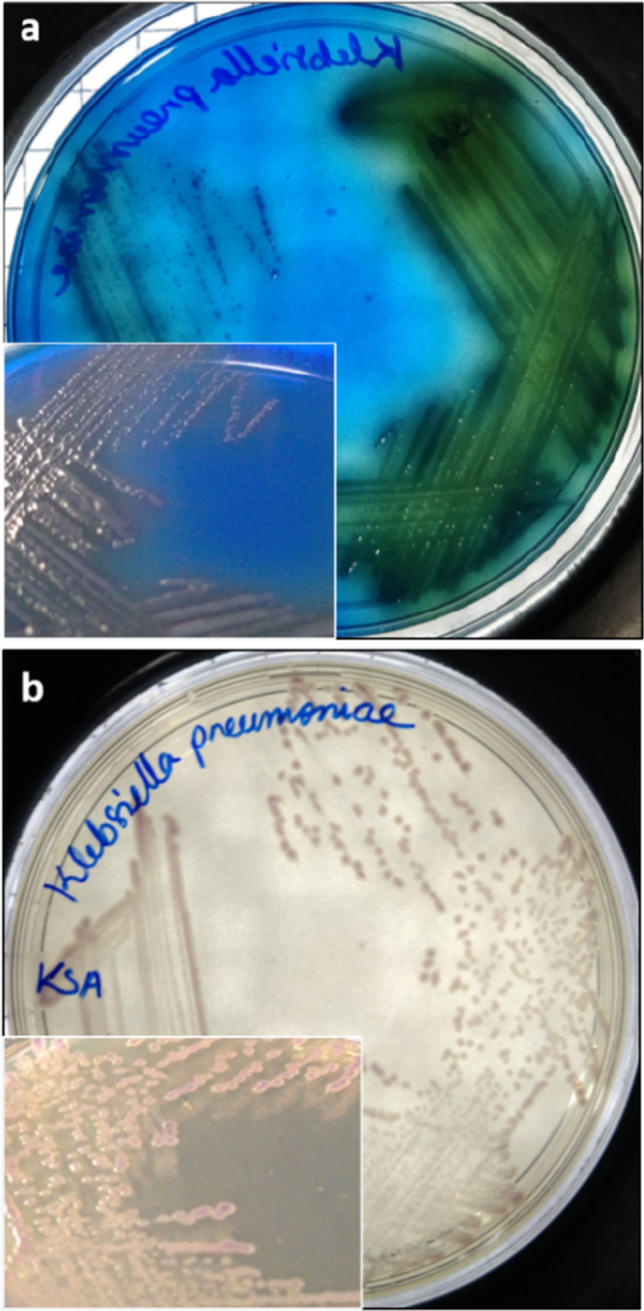
Cultural characteristics of *K. pneumoniae* on KBA and KSA. Colonies of *K. pneumoniae* (MTCC 3384) in **a** KBA and **b** KSA medium after 16 ± 2 h of incubation at 37 °C appear green and light purple, respectively

**Fig. 2:**
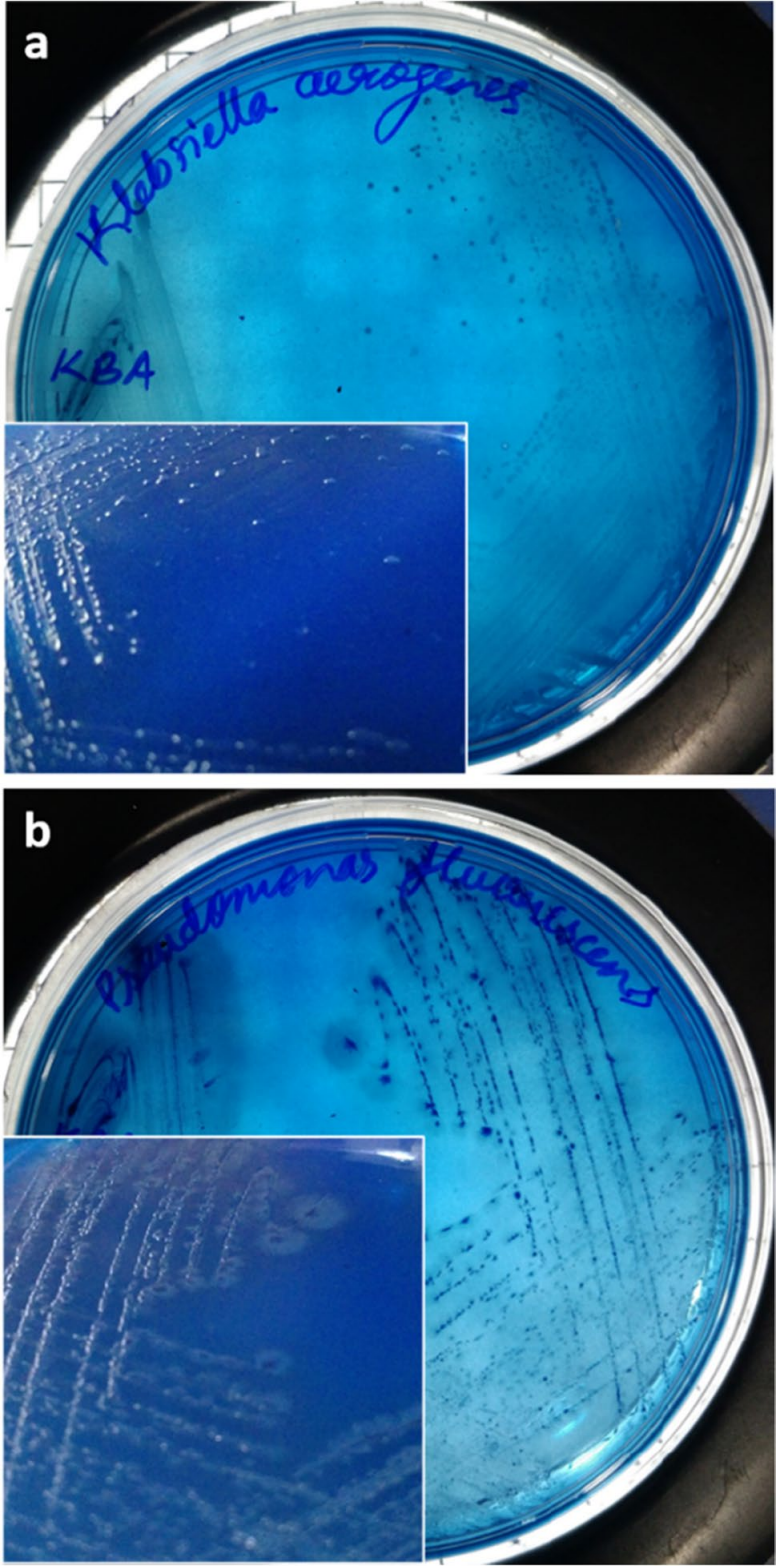
Cultural characteristics of *K. aerogenes* and *P. fluorescens* in KBA medium. The differential nature of the KBA medium can be inferred from the growth of **a** *K. aerogenes* and **b** *P. fluorescens* with different colony morphologies and colours in KBA

Freshly prepared KBA medium appeared deep blue due to methylene blue. The medium allowed for the selective growth of the *Klebsiella* spp. — *K. pneumoniae*, *K. quasipneumoniae*, *K. variicola*, and *K. aerogenes* over other bacterial cultures*.* Pronounced growth of *Klebsiella* spp. was observed after 16 ± 2 h of incubation at 37 °C. The media colour surrounding the *Klebsiella* spp. colony changed from blue to dark green [Fig. 1]. The colony appeared mucoid with a dark green sheen which increased upon incubation at 37 °C for 24 h [Fig. 1]. Not all the *Klebsiella* spp. showed a dark green sheen, *K. aerogenes* had a translucent mucoid colony at 24 h but developed a faded green colouration after incubation of 48 h [Fig. 2a]. When colonies of *Klebsiella* spp. were closely spaced, the media surrounding the bacterial colonies had a dark green colour. In KSA, the colonies of all the species of *Klebsiella* had the same purple magenta colour.

### Selective and differential nature of the KBA agar

**Fig. 3:**
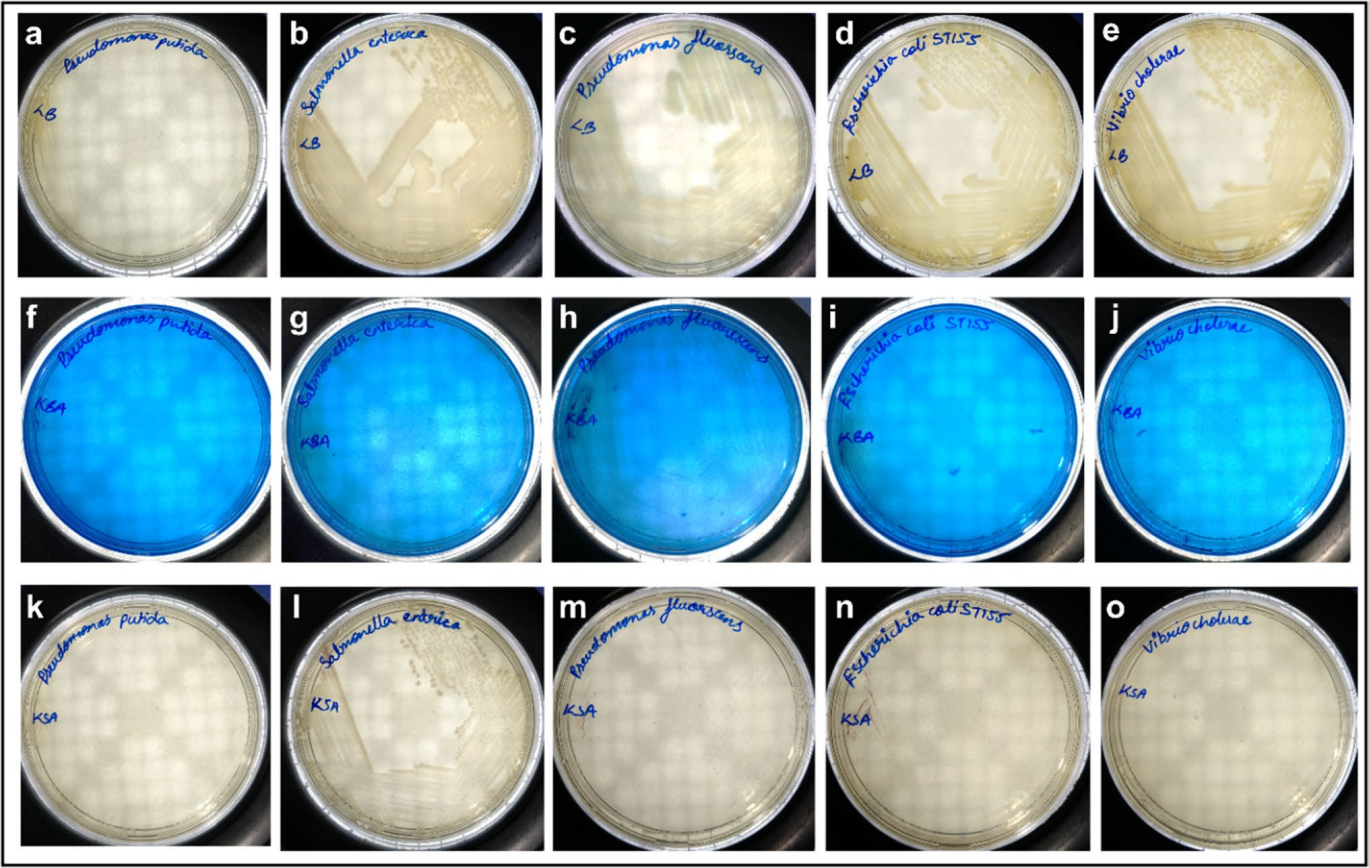
Bacterial growth profile on LB, KBA, and KSA. The difference in the bacterial growth profile in the LB (**a** to **e**), KBA (**f** to **j**), and KSA (**k** to **o**) media plates. The figures **a**, **f**, and **k** correspond to *P. putida*; **b**, **g**, and **l** correspond to *S. enterica*; **c**, **h**, and **m** correspond to *P. fluorescens*; **d**, **i**, and **n** correspond to *E. coli* ST155; and **e**, **j**, and **o** correspond to *V. cholerae*

**Fig. 4:**
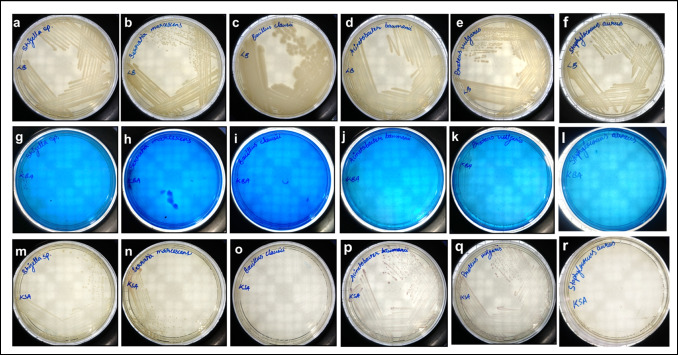
Bacterial growth profile on LB, KBA, and KSA. The difference in the bacterial growth profile in the LB (**a** to **f**), KBA (**g** to **l**), and KSA (**m** to **r**) media plates. The figures **a**, **g**, and **m** correspond to *Shigella* spp.; **b**, **h**, and **n** correspond to *S. marcescens*; **c**, **i**, and **o** correspond to *Bacillus* spp.; **d**, **j**, and **p** correspond to *A. baumanii*; **e**, **k**, and **q** correspond to *P. vulgaris*; and **f**, **l**, and **r** correspond to *S. aureus*

**Fig. 5:**
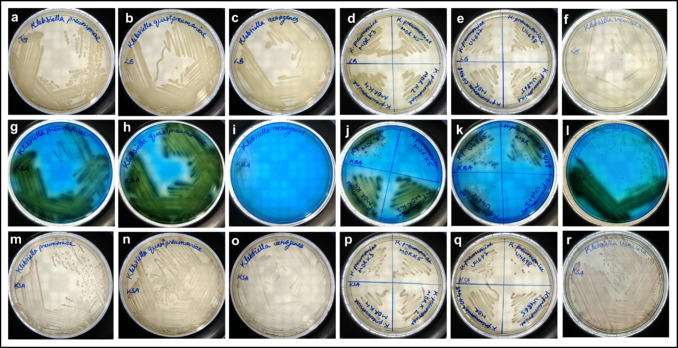
Bacterial growth profile on LB, KBA, and KSA. The difference in the bacterial growth profile in the LB (**a** to **f**), KBA (**g** to **l**), and KSA (**m** to **r**) media plates. The figures **a**, **g**, and **m** correspond to *K. pneumoniae* (MTCC 3384); **b**, **h**, and **n** correspond to *K. quasipneumoniae*; **c**, **i**, and **o** correspond to *K. aerogenes*; **d**, **j**, and **p** correspond to MDR strains of *K. pneumoniae* K2, K3, K4, and K5 respectively; **e**, **k**, and **q** correspond to clinical strains of *K. pneumoniae* U4677, U4698, U4865, and OF9168 respectively; and **f**, **l**, and **r** correspond to *K. variicola*

*K. pneumoniae*, *K. aerogenes*, *K. quasipneumoniae*, *K. variicola*, and all MDR strains of *K. pneumoniae* showed a well-defined growth in the KBA medium (Fig. 5). All the colonies were green in colour, except for *K. aerogenes*, *K. pneumoniae* MDR K5, and *K. pneumoniae* U4677 which had a translucent mucoid colony (Fig. 5h, i, and j). KBA medium was able to repress the growth of *Shigella* spp., *S. marcescens*, *Bacillus* spp., *S. aureus* [Fig. 4g, h, i, and l], *P. putida*, *S. enterica*, and *V. cholerae* [Fig. 3f, g, and j)] while there was a scanty growth for *A. baumannii*, *P. vulgaris* [Fig. 4j and k], *E. coli* ST155 and *P. fluorescens* [Fig. 3h and i]. *P. fluorescens*, when incubated for an extended period of 48 h, showed a prominent and distinguishable blue-coloured colony formation [Fig. 2b]. *E. coli* ST155, upon incubation up to 48 h, did not show any significant increase or change in colony morphology or growth.

*K. pneumoniae*, *K. aerogenes*, *K. quasipneumoniae*, *K. variicola*, and all MDR strains of *K. pneumoniae* [Fig. 5m, o, n, r, p, and q] grew in the KSA with a light purple colour. However, we observed that *P. vulgaris* and *A. baumannii* [Fig. 4p and q] showed the same purple pigmentation when cultured in KSA. *E. coli* ST155 [Fig. 3n] showed a scanty growth after incubation for 24 h. The colonies of *S. enterica* [Fig. 3l] and *Shigella* spp. [Fig. 4m] grew in the KSA plate as milky white colonies. *S. marcescens* [Fig. 4l] colonies appeared light pink at 24 h, changing to a brighter shade after 48 h. LB being a nutritionally rich media supported the growth of all cultures and showed their respective colony morphologies as expected [Fig. 3a–e; Fig. 4a–f; and Fig. 5a–f].

### Selective isolation of Klebsiella spp. from synthetic sewage by KBA

**Fig. 6:**
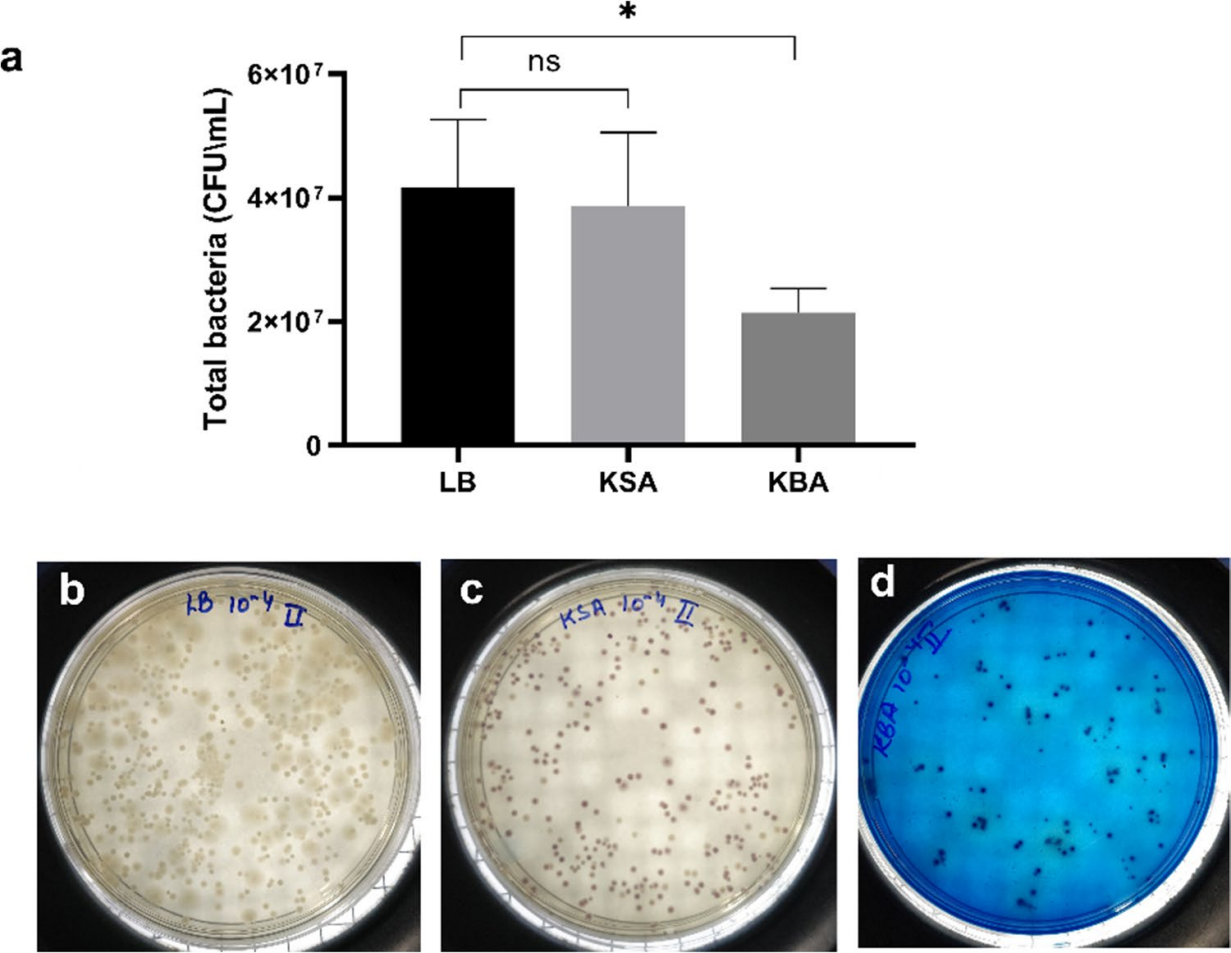
Selective isolation of *Klebsiella* spp. on KBA from a heterogenous population in synthetic sewage. **a** Relative differences in the bacterial population on LB, KSA, and KBA were estimated and plotted (*p* ≤ 0.05) (Graph pad prism 9.0). The images **b**, **c**, and **d** correspond to the LB, KSA, and KBA media plates from which the colony count was estimated

The difference in the bacterial count of simulated synthetic sewage when plated onto the LB, KSA, and KBA media was determined. KSA had a lesser bacterial count of 3.87 × 10^7^ CFU/mL than LB, with a colony count of 4.17 × 10^7^ CFU/mL, while KBA being a more selective medium of the three had only 2.14 × 10^7^ CFU/mL [Fig. 6a]. Selectivity of the medium could be seen from the plates of LB, KSA, and KBA, where in apart from the decrease in the colonies in KBA, the colonies of the *Klebsiella* spp. are growing with their characteristic green colouration. The selective nature of the KBA was further confirmed by colony-based multiplex PCR using primers specific for *K. pneumoniae* and *K. quasipneumoniae*. The PCR result confirms that the colonies in the KBA media are of *Klebsiella* spp. [Fig. S1e, f, and g]. Even though the colonies growing in LB and KSA have *Klebsiella* spp., it also supports the growth of other bacteria present in the simulated sewage [Fig. S1a, b, c, and d].

### Increased selective nature of KBA over KSA

**Fig. 7:**
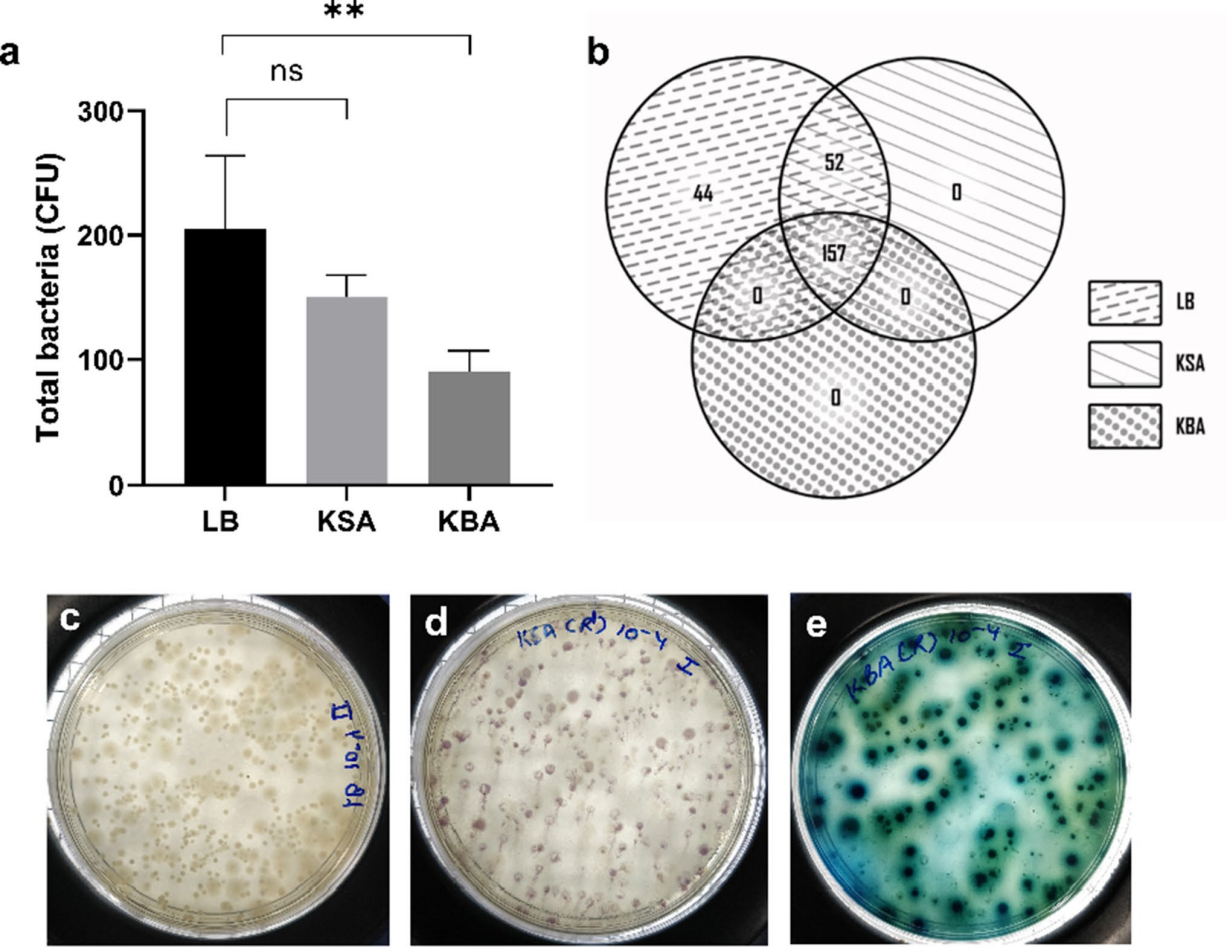
Replica plating for analysing the selective nature of KBA. **a** Relative differences in the bacterial population on KSA, and KBA, when replica plated from LB, were estimated, and plotted (*p* ≤ 0.05) (Graph pad prism 9.0). **b** Venn diagram indicating unique and shared colonies on LB, KSA, and KBA. **c**, **d**, and **e** correspond to the LB, KSA, and KBA media plates from which the colony count was estimated

The replica plating technique was used to demonstrate the selective nature of the KBA medium over the KSA medium. LB medium had 253 CFU, of which only 209 CFU grew in the KSA medium when transferred [Fig. 7a]. While in the KBA, out of 253 CFU, only 157 CFU grew when transferred [Fig. 7a]. The Venn diagram shows the relative growth comparison of the colonies in LB, KSA, and KBA [Fig. 7b]. All the colonies in KBA medium were growing in KSA, but not all colonies found in KSA were growing in KBA but were present in the primary LB plate. Thus, it could be inferred that KBA is more selective than KSA, which was further confirmed using the one-step multiplex colony PCR.

## Discussion

Sewage is a hotspot for antibiotic-resistant bacteria, and there is a need for routine monitoring of the sewage to assess the prevalence of bacterial pathogens, for example, *Klebsiella* spp. The genus is fast gaining attention for its resistance to last-resort treatment (carbapenem antibiotics) (Kumar et al. 2018), which has led us to formulate a new differential and selective media for their isolation from environmental samples. The contribution of environmental reservoirs to the ever-increasing healthcare-associated *Klebsiella* infections and nosocomial outbreaks is increasing linearly (Perez-Palacios et al. 2021b). The effective monitoring and mitigative strategies demand discrimination of *Klebsiella* spp. falling into different cohorts. KpSC is a pathogenically important group that includes *K. pneumoniae*, *K. quasipneumoniae*, and *K. variicola.* The other *Klebsiella* spp., viz., *K. aerogenes*, *K. oxytoca*, *K. indica*, and *K. terrigena*, to name a few, constitute the other group.

The KSA medium has often been used to isolate *Klebsiella* spp. from diverse sources (Divakaran et al. 2019; Varshney et al. 2021). However, our preliminary study suggested that KSA supports the growth of *P. vulgaris* and *A. baumannii* in the same way as that of *Klebsiella* spp. [Fig. 4p and q], and it was difficult to distinguish them from each other. To tackle this problem, we ventured to develop a new selective medium that would support the growth of *Klebsiella* spp. while suppressing the growth of other gram-negative and most gram-positive bacteria. The new medium enabled the differentiation of the colonies of *Klebsiella* spp. belonging to the KpSC with a characteristic green colouration [Fig. 5g–l].

The availability of different carbon sources supporting the growth of *Klebsiella* spp. was investigated in the study by van Kregten et al. (1984), Grimont and Grimont (2015), and Kumar and Park (2018), which was used as references for selecting the carbon source in the KBA medium. Both lactose and glycerol are known to support the growth of *Klebsiella* spp., hence were analysed as the sole carbon source in the KBA medium. It was observed that when compared to lactose, the selectivity improves with the inclusion of glycerol [Figs. 3, 4, and 5]. On the other hand, lactose supported apart from *Klebsiella* spp., growth of *P. vulgaris*, *E. coli* ST155, *A. baumanii*, and *S. enterica* with the same characteristic green colouration as that of the *Klebsiella* colonies after 24 h of incubation. Glycerol is also known to support the growth of *Clostridium pasteurianum*, *Clostridium butyricum*, and *K. aerogenes* (Valan Arasu et al. 2011). However, methylene blue in the medium inhibited the growth of gram-positive bacteria like *Clostridium* spp. and *Bacillus* spp. [Fig. 4i]. The *Klebsiella* spp. metabolises the glycerol present in the medium, producing acid that precipitates the dye onto the growth surface, imparting a characteristic green sheen to the bacterial colonies. The KBA medium, being differential in nature, was able to differentiate between the growth of *K. aerogenes* from *K. pneumoniae* and *K. quasipneumoniae*, and *K. variicola* (KpSC complex)*.* The colonies of *K. aerogenes* appeared as translucent mucoid colonies [Fig. 2a], which was different from the colonies of *K. pneumoniae* [Fig. 1a], *K. quasipneumoniae* [Fig. 5h], and *K. variicola* [Fig. 5l]. It is pertinent to note that apart from *Klebsiella* spp., *A. baumannii*, *P. vulgaris* [Fig. 4j and k], *E. coli* ST155, and *P. fluorescens* [Fig. 3h and i] grew in the KBA medium scantily. Except for *P. fluorescens*, which grew with a blue colouration [Fig. 2b], none of the other organisms showed any increased growth after 48 h. We observed during the formulation of the medium that an optimal concentration of NaCl improved the differential nature of the media. From a range of NaCl concentrations of 0.1 to 0.8%, 0.6% was found to be the optimal concentration.

A 0.15% concentration of bile salt is known to support the growth of gram-negative bacteria while inhibiting most of the gram-positive (Cremers et al. 2014), hence was used in the KBA medium to make it selective. The rationale for including tryptophan in the medium was to promote the growth of *Klebsiella* spp. amongst which many are indole positive, and the remaining can use tryptophan as a carbon or nitrogen source. The pH of the media is 7.20 ± 0.2, and the presence of K_2_HPO_4_ and KH_2_PO_4_ acts as a buffering component in the medium. MgSO_4_ was added as a micronutrient which acts as a cofactor for enzymatic reactions. Moreover, all these features make the media non-conducive for competing organisms that exhibit no growth after 24 h of incubation. The composition of the chromogenic mixture in the KSA medium is proprietary and incurs high cost. KBA’s composition is well defined without any complex mixture, containing only methylene blue, tryptophan, and bile salt as the selective and differential components. Hence owing to its readily available components, the medium can be easily formulated and rampantly used in laboratories.

KBA medium was able to selectively isolate *Klebsiella* spp. from simulated synthetic sewage augmented with bacteria (total bacterial count of 4.17 × 10^7^ CFU/mL) of different genera, including nine strains of *K. pneumoniae *[Fig. 6a and d]. Although KSA supported the growth of *Klebsiella* spp., it also favoured the growth of other bacteria (3.87 × 10^7^ CFU/mL). It was confirmed using a one-step multiplex colony PCR with primers specific for *K. pneumoniae* and *K. quasipneumoniae* [Fig. S1a to g]. The primers SHV-f and OKP-f are specific and target the chromosomal class A β-lactamase gene *bla*_SHV_ and *bla*_OKP_ of *K. pneumoniae* and *K. quasipneumoniae* [Table 1] respectively. The reverse primer DeoR-r is derived from a gene coding for an ATPase that is part of the stable bacterial genome and flanks the respective *bla*_SHV_ and *bla*_OKP_ genes (Fonseca et al. 2017). One-step multiplex colony PCR showed that KBA is relatively more selective than KSA.

Replica plating of LB plate onto KSA and KBA showed that KBA was more selective for *Klebsiella* spp. than KSA medium. Out of the 253 colonies [Fig. 7a and b], only 157 colonies grew on the KBA medium, while 209 colonies grew on the KSA medium, out of which 52 colonies were unique to KSA but not to KBA. These results indicated the selective nature of the medium.

KBA owing to its proven characteristics, namely facile formulation, improved selectivity, differential nature, and cost-effectiveness, could enhance its potential to be used in environmental settings such as wastewater. Furthermore, elaborate field testing and exploration of its commercial viability can lead to efficient translation for monitoring, surveillance, and basic research applications.

## Supplementary information

Below is the link to the electronic supplementary material.

Supplementary file1 (PDF 198 KB)

## Data Availability

The authors agree in principle to make the data presented in the article to be available in freely accessible resources.

## Declarations

The authors have no commercial interests in any material discussed in this article.
