# Supplementary material for: A novel and improved selective media for the isolation and enumeration of Klebsiella species
Source: Appl Microbiol Biotechnol. 2022 Nov 16;106(24):8273–84. doi: 10.1007/s00253-022-12270-w (PMC9726774; doi:10.1007/s00253-022-12270-w)
Supplement: Supplementary file 1 — Supplementary file1 (PDF 198 KB) [file 253_2022_12270_MOESM1_ESM.pdf]

Supplementary Material

A novel and improved selective media for the isolation and enumeration of *Klebsiella* species

Applied Microbiology and Biotechnology

Megha Prasad[1], Sindhu K Shetty[1] Bipin G Nair[1], Sanjay Pal[1]*, Ajith Madhavan[1]*

Affiliation 1. School of Biotechnology, Amrita Vishwa Vidyapeetham, Kerala, 690525, India

Corresponding authors – Dr. Ajith Madhavan, Email: ajithm@am.amrita.edu; ORCID: 0000-0002-4519-0851; Dr. Sanjay Pal, sanjaypal@am.amrita.edu; ORCID: 0000-0002-2012-0734

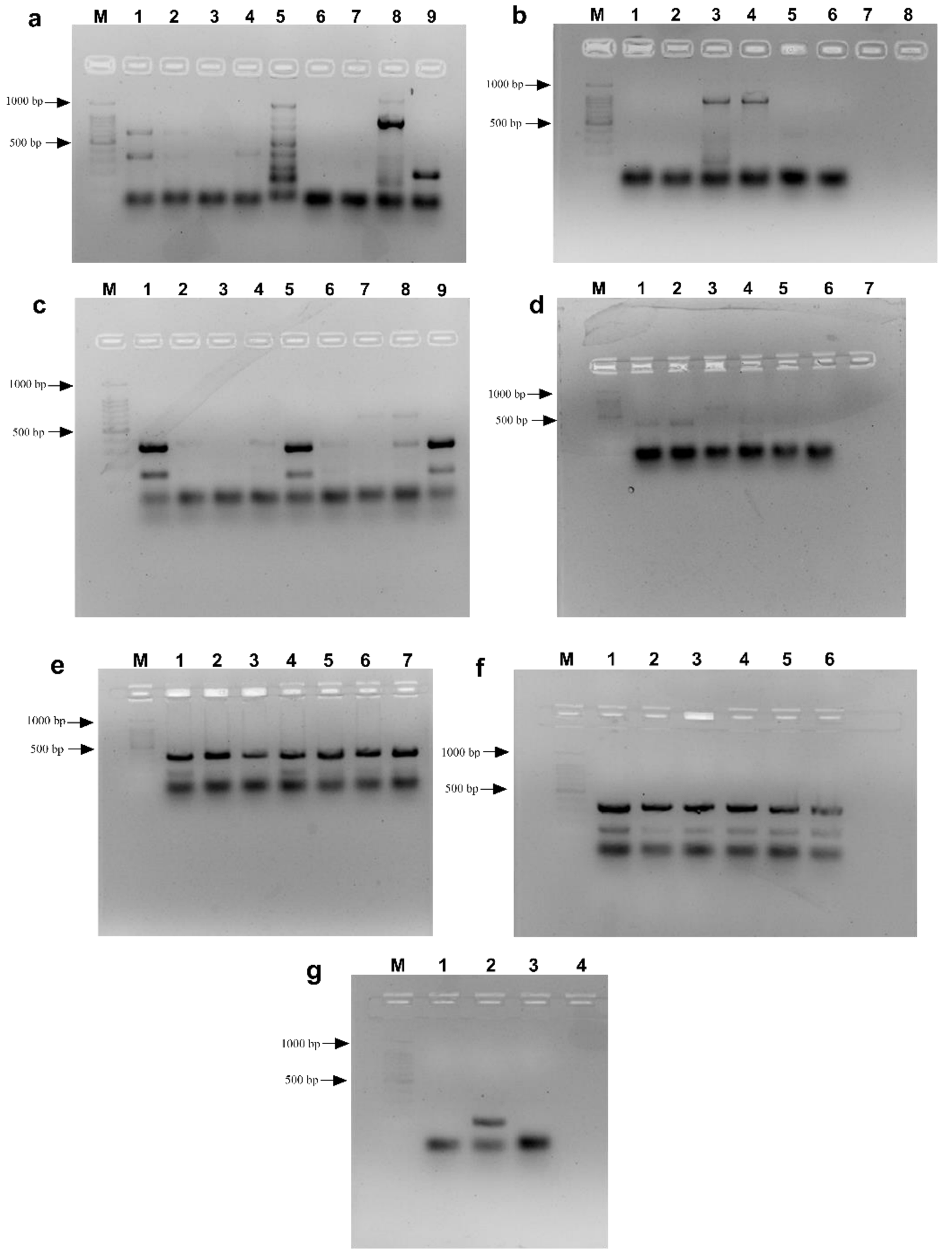

Fig. S1 Multiplex PCR amplification using the primer set mentioned in Table 1 for colonies in **a)** lanes 1 to 9 & **b)** lanes 1 to 6 from LB medium plate; **c)** lanes 1 to 9 & **d)** lanes 1 to 6 from KSA medium plate; and **e)** lanes 1 to 7 **f)** lanes 1 to 6 & **g)** lanes 1 to 2 from KBA media plate, lane 3 corresponds to the non-template control. PCR samples were subjected to electrophoresis on 2.5% agarose gel. Lane M is the DNA molecular size marker of 100 bp DNA ladder (Origin Diagnostics, and Research Pvt. Ltd.). The amplicon of 995 bp size corresponds to the $\mathit{bla}_{\mathrm{SHV}}$ gene – specific for *Klebsiella pneumoniae* while the amplicon of 348 bp size corresponds to the $\mathit{bla}_{\mathrm{OKP}}$ gene – specific for *Klebsiella quasipneumoniae.*
